# Supplementary material for: Arbuscular Mycorrhizal Fungi Enhance the Insecticidal Activity of Annona muricata L. Leaves
Source: Plants (Basel). 2025 Nov 17;14(22):3501. doi: 10.3390/plants14223501 (PMC12656605; doi:10.3390/plants14223501)
Supplement: Supplementary file 1 [file plants-14-03501-s001.zip › plants-3914384-supplementary.pdf]

Supplementary material

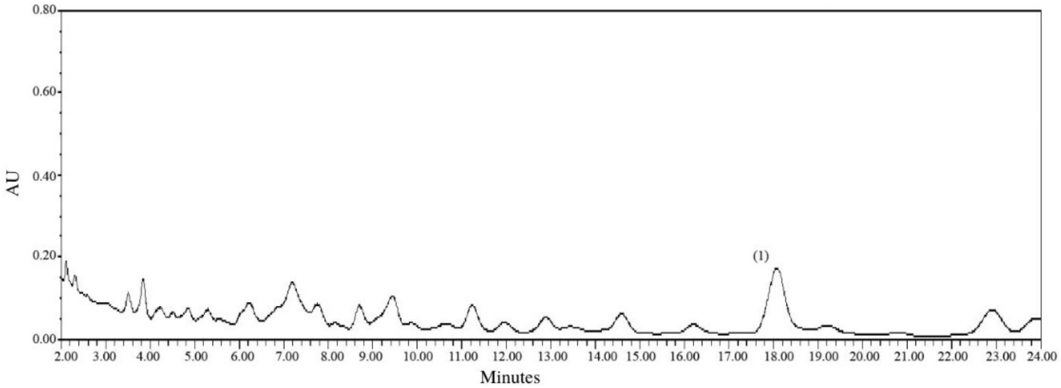

Figure S1: Chromatogram of *A. muricata* leaf methanolic extract. C18 5 µm (4.6 mm x 150 mm) column; mobile-phase acetonitrile and water 70:30 (isocratic system) with a flow rate of 1.20 mL/min; UV detection λ = 210 nm; injection volume, 15 µL. *A. muricata* plants colonized by *Funneliformis mosseae*.

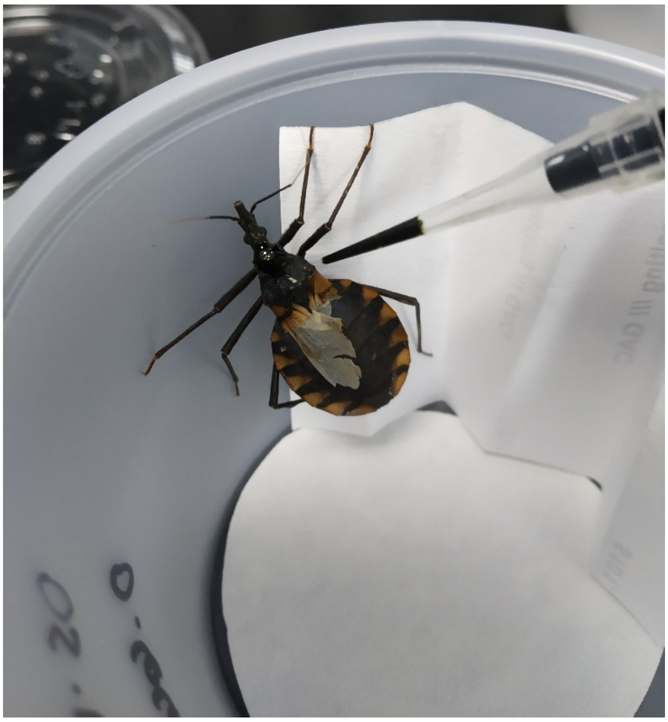

Figure S2: Application of ethanolic leaf extracts of *Annona muricata* to adult *Triatoma pallidipennis* specimens. Extracts were prepared at concentrations of 400 and 800 mg·mL^{-1} in 100% DMSO and applied to the pronota of adult insects (10 males and 10 females per treatment) using a micropipette. The treated insects were placed individually in 250 mL polyethylene jars containing filter paper at the bottom and maintained at 26 °C and 60–70% relative humidity for 30 days. Mortality was recorded every three days.

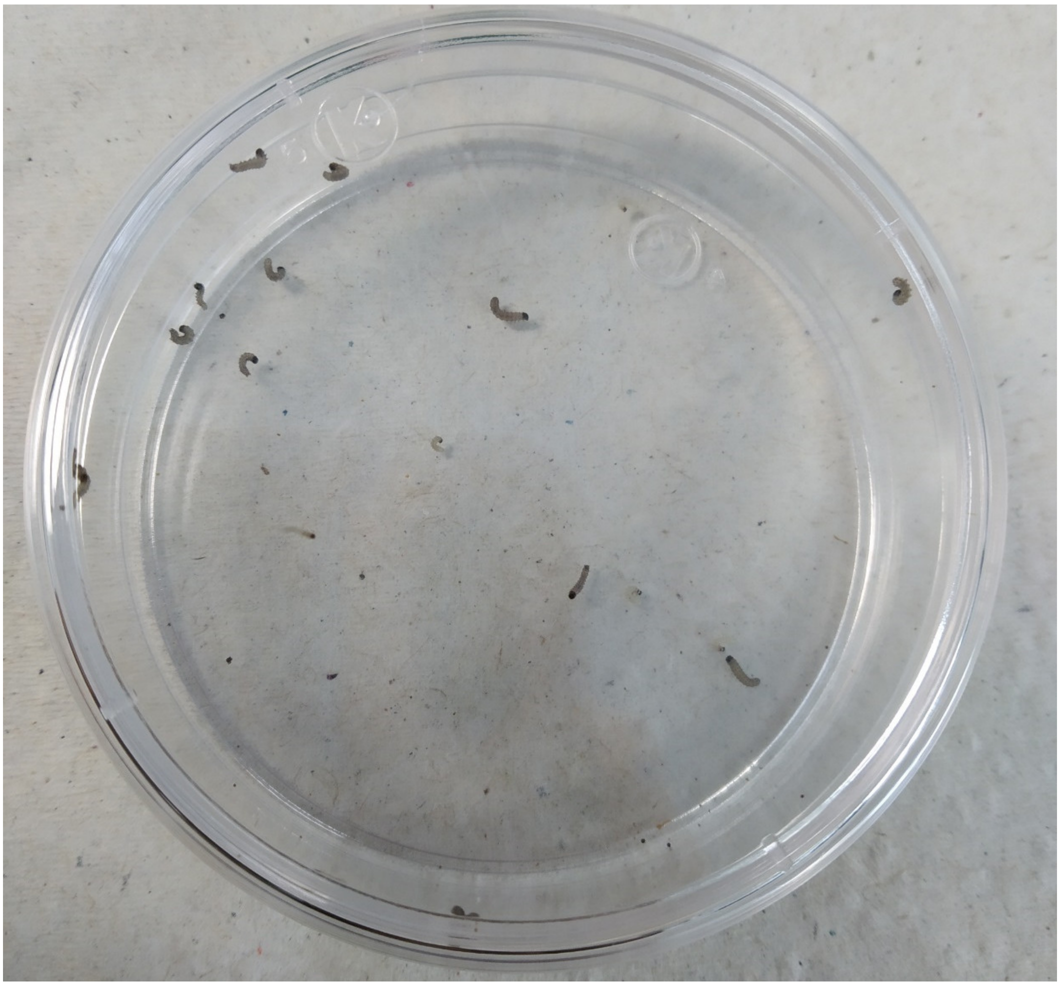

Figure S3: Second-instar larvae of *Spodoptera frugiperda* used in the bioassay. Larvae were individually placed in wells containing diet plugs corresponding to each treatment and maintained under controlled laboratory conditions (26 °C, 60–70% relative humidity, and 12:12 h light–dark photoperiod).

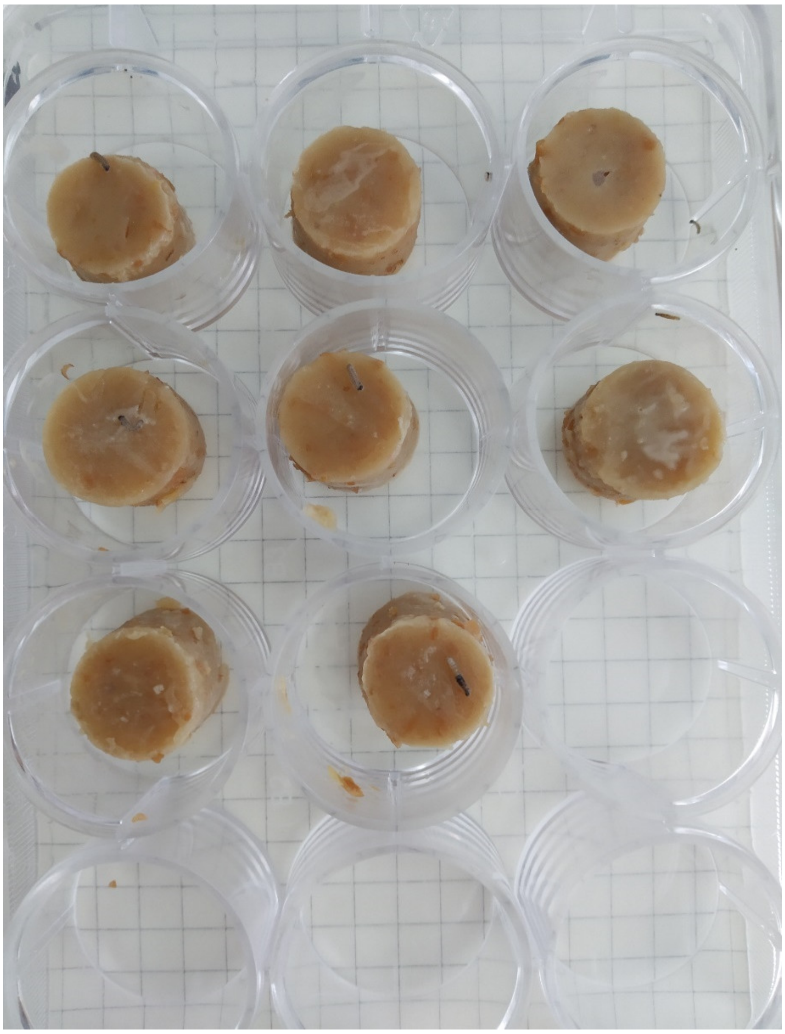

Figure S4: Second-instar larvae of *Spodoptera frugiperda* placed on diet plugs corresponding to the positive control treatment (Palgus®, 0.066 μL·mL^{-1}). Each larva was confined individually in a 12-well tray and maintained under controlled laboratory conditions (26 °C, 60–70% relative humidity, and a 12:12 h light–dark photoperiod) during the bioassay.
